# Notes and News

**Published:** 1886-10-02

**Authors:** 


### Notes and News.

Collected and Communicated.

Prince Albert Victor has accepted an invitation by the Committee of the new Victoria Hospital at Burnley, to perform the opening ceremony. The date fixed is the 13th inst. The new edifice is now well-nigh ready.

"A town without a hospital," said Cardinal Manning at the recent opening of the new St. Helen's Hospital, "is like the sky without the sun." The metaphor was just and well chosen. The poor of a past generation too often regarded our hospitals with aversion. The change which has come about is one of happy augury.

In England and Wales at the present moment there are rather over 80,000 persons of unsound mind, of whom 71,000 odd are paupers. The Blue-Book, from which we take these figures, contains a ray of hope, however, as the total increase of the year was only 452 cases, an increase which is considerably below that of the past twenty-five years. A temperance reformer states, without giving evidence of the fact, that probably at least one-half of the lunacy to be found in England has arisen directly or indirectly through over-indulgence in alcoholic drinks.

The importance of convalescent hospitals is recognised on all hands, but up to the present time the want has not been adequately met. Active steps are, however, now being taken for carrying out the scheme for the erection of a London Convalescent Hospital, inaugurated at the Mansion House, some twelve months ago. The Council, of which Earl Cowper is chairman, and Sir Spencer Wells vice-chairman, comprises some very influential names, and already a large amount of pecuniary support has been promised.

So heavy are the demands upon the resources of our hospitals that patients are necessarily discharged at the earliest possible moment, and consequently before their health has been anything like completely restored. With the very poor this stage of convalescence is a very critical period. The poor working man or woman can afford themselves neither the luxury of rest nor appropriate diet. No sooner does the poor patient reach his home than he must needs gird on the yoke of work. This sudden transition is but too likely to undo the good which has been done him. More convalescent hospitals are wanted to help to carry the poor bread-winner over this trying time, and would send him home restored to health.

Many of our hospitals have considered this important question, and a few have established convalescent branches, but the present undertaking is the first aiming at organised assistance among our hospitals generally. It is proposed that the administration of this projected Convalescent Hospital shall be carried on with the assistance of members of the medical profession associated with all the chief London hospitals. Convalescents, where they are in a position to pay something towards their maintenance, are not to be pauperised by being maintained entirely at the expense of the hospital, though no charge will be made to those who are really necessitous.

Surgeon-Major Evatt has had for some time past a plan for organising a corps of volunteer female nurses, who would be ready at any given moment to join the army hospitals in the field. Such a corps as he proposes would be supplementary to the existing staffs of nursing sisters now employed in the army hospitals, and he believes that such a body might be made to form the basis of a first definite attempt to incorporate the nursing profession. Many of the objectors to Dr. Evatt's scheme will heartily agree with him that the profession of the nurse ought to possess as distinct an autonomy and self-government as is enjoyed in most organised professions.

Dr. Evatt's proposals naturally suggest the inquiry, "Is there need for a Society for the protection of the nursing community?" It is only during the past few years that the art of nursing has attained any footing as a distinct and honourable profession, a profession which calls into active play all the higher and more humanising qualities of human nature. Nursing, it is true, has now been systematised, and schools for training have been established, but at present these institutions are so many scattered and disunited elements. The formation of a Sectional Committee on Nursing and domestic management in connection with the Hospitals Association, under the presidency of Mrs. Wardroper, should prove the means of banding in one great sisterhood all who have dedicated their lives to a noble profession. We hope arrangements may be made to take care of the sick and decayed members of the nursing profession, and to render aid to them in many other directions.

There is probably no position which, rightly understood, tends to develop the best faculties of our nature, like responsible office in a hospital or asylum. The superintendent has the authority of a kinglet, and upon his capacity and conduct the efficient working of the whole concern depends. These truths are gradually coming to be realised by the governing bodies, and the tendency of the day is to add to the responsibility of the chief officials and to increase their rate of remuneration.

---

The public also are becoming alive to the valuable training which is here afforded for men of capacity. If a superintendent or secretary makes the institution under his control to prosper and advance by "leaps and bounds," his services are soon sought for by those who have openings for really capable men. Thus Mr. J. W. Waldron, who did such good work at King's College Hospital, which was never more prosperous than under his management, was promoted to the responsible post of secretary to the Union Club, and has now been selected for the coveted and honourable position of under treasurer of the Middle Temple. Mr. J. S. Wood's promotion from the secretaryship of the Chelsea Hospital for Women to the managing directorship of the Kensington Agricultural Hall has given general satisfaction, and is another proof of the rewards which are sure to come to the really capable men in the hospital field.

---

Asylum superintendents, too, follow the same rule. The bad days, when inexperienced men were selected as Commissioners in Lunacy, cannot long continue, and for the future public opinion will insist that the Commissioners shall be selected from the most capable of the medical superintendents throughout the country. To secure the proper working of such a system as this, the salaries paid to the Commissioners may have to be reconsidered, as the causes we are considering have made the remuneration of asylum superintendents more in proportion to the responsibilities undertaken, and in one division of the country the maximum income per annum of the superintendents at the majority of the asylums has been raised of late from £800 to £1,000, exclusive of house and allowances. This is as it should be, and public bodies can make no more economical expenditure than liberal salaries as the reward of loyal and devoted service in the cause of suffering humanity.

---

Marrying and giving in marriage has always been a rock ahead in the training schools for nurses. It speaks well for the discipline and for the good sense of all concerned that these useful institutions have been so free from scandal. What more natural than that association in caring for and tending the suffering should sometimes result in the growth of sympathetic feeling? At the same time it cannot be doubted that any woman who takes up nursing without a serious liking for the work, and a determination to devote her whole energies to it, is a person to be avoided by the hospital authorities. The woman with ulterior designs at the start, if any such there be, is one of whom little that is good can be expected or said. She abuses the field of charity by her presence, and is on every ground to be avoided and ostracised by those who love nursing.

---

The matron's lot is not always, if at all, a happy one. It sometimes happens, as in a recent case, that ladies who have had no experience in the management of large institutions, beyond that which is to be obtained as sister of a large special ward, find themselves elected as superintendents with full authority over the nursing and domestic departments. What wonder is there if in such cases failure follows and nursing is discredited? All nursing schools to where ladies are for trained responsible office ought to provide in their system, and to show by their certificates, that an opportunity has been given for fulfilling the duties of lady superintendent, and that the holder of the certificate has shown marked, fair, or moderate talent for their efficient discharge.

---

The public cannot be too often reminded, when in need of the services of a trained nurse, that they have no guarantee of knowledge or efficiency, unless the nurse has a certificate showing that she has been trained for at least a year at a recognised nursing school. Testimonials from individuals are never to be relied upon as evidence of training or capacity, and attention to this caution will save the public much worry and danger. Besides, the opportunities for training are now so great as to make it not only desirable, but an act of justice, to give the preference to trained certificated nurses.

---

It is mainly due to Mr. Sexton, M.P., that Parliament has been induced to grant a sum of £500 to the Belfast Royal Hospital, in consideration of the exceptional demands made upon the resources of that institution during the recent riots. Before these troubles began, the hospital was in debt, and the disturbances of the past few weeks have resulted in some 370 patients being thrown upon the hospital, entailing an expenditure of something like £800. Mr. Sexton has shown himself a true friend to the Belfast Royal Hospital.

